# CRISPR-Cas9: bridging the gap between aging mechanisms and therapeutic advances in neurodegenerative disorders

**DOI:** 10.3389/fncel.2025.1681891

**Published:** 2025-10-16

**Authors:** Anas Shamsi, Mohammed Alrouji, Othman AlOmeir, Syed Tasqeruddin, Khuzin Dinislam, Azna Zuberi

**Affiliations:** 1 Centre of Medical and Bio-allied Health Sciences Research, Ajman University, Ajman, United Arab Emirates; 2 Department of Medical Laboratories, College of Applied Medical Sciences, Shaqra University, Shaqra, Saudi Arabia; 3 Department of Clinical Pharmacy, College of Pharmacy, Shaqra University, Shaqra, Saudi Arabia; 4 Department of Pharmaceutical Chemistry, College of Pharmacy, King Khalid University, Abha, Saudi Arabia; 5 Department of General Chemistry, Bashkir State Medical University, Ufa, Russia; 6 Department of Obstetrics and Gynecology, Northwestern University, Chicago, IL, United States

**Keywords:** CRISPR-Cas9, gene editing, neurodegenerative diseases, Alzheimer’s disease, Parkinson’s disease, Huntington’s disease, amyotrophic lateral sclerosis (ALS), aging

## Abstract

Neurodegenerative diseases such as Alzheimer’s, Parkinson’s, Huntington’s, ALS, and spinocerebellar ataxia are becoming more prevalent as populations age, posing major global health challenges. Despite decades of research, effective treatments that halt or reverse these conditions remain elusive. Aging is the most significant risk factor in the development of these diseases, intertwining with molecular processes like DNA damage, mitochondrial dysfunction, and protein aggregation. Recent advances in gene-editing technologies, particularly CRISPR-Cas9, are beginning to shift the therapeutic landscape. This revolutionary tool allows for precise correction of genetic mutations associated with neurodegeneration, offering the potential for disease modification rather than symptom management alone. In this review, we explore how CRISPR-Cas9 is being leveraged to target key genes implicated in various neurodegenerative conditions and how it may overcome barriers posed by aging biology. We also examine the delivery systems and safety challenges that must be addressed before clinical application. With continued progress, CRISPR-Cas9 could mark a turning point in our ability to treat or even prevent age-related neurological decline.

## Introduction

A class of diseases known as neurodegenerative diseases is defined by a steady deterioration in the composition and functionality of neurons, which eventually results in neuronal death (Rahimi et al., 2024; Gitler et al., 2017; Checkoway et al., 2011; Wilson et al., 2023; Gan et al., 2018). These medical conditions are extremely incapacitating and have a major effect on motor and cognitive abilities. While examining the literature surveys and medical reports, some of the most common neurodegenerative diseases discussed in them are Alzheimer’s disease (AD), which affects the most significant number of patients, while the second is Parkinson’s disease (PD) (Gitler et al., 2017; Checkoway et al., 2011; Wilson et al., 2023; Gan et al., 2018). These diseases are predicted to become more common as life expectancy rises globally, necessitating immediate prevention and therapeutic measures. About 50 million individuals across the world suffer from dementia, a severe neurological disease, and by 2050, that figure is expected to rise to 130 million. With 60%–70% of cases, AD is the most prevalent type of dementia, whereas PD is the second leading cause of such devastating diseases, impacting over 6 million individuals worldwide (Pang et al., 2022; DeTure and Dickson, 2019). The primary symptoms that appear in patients are the episodic loss of memory, which is followed by cognitive dysfunction, language issues, and visuospatial impairments. Amyloid-β (Aβ) plaque buildup and tau-containing neurofibrillary tangles are its pathological hallmarks. These aggregates, which are caused by genetic mutations and other reasons, start to form decades before dementia symptoms appear. With 20% of women and 10% of males acquiring AD, women are disproportionately affected (Silva et al., 2019). Bradykinesia, rigidity, resting tremor, and abnormalities in gait are some of the signs of PD, a movement disorder (DeMaagd and Philip, 2015). Lewy bodies (hard protein clumps linked to many other neurodegenerative diseases) and Lewy neurites (faulty thread-like filamentous structures), which are mainly found in dopamine-producing neurons, are the result of α-synuclein aggregation. Constipation and sleep issues are examples of non-motor symptoms that frequently appear years before motor problems (Pang et al., 2022; DeMaagd and Philip, 2015). An uncommon genetic condition known as Huntington’s disease (HD) is brought on by an increase in CAG trinucleotide repeats in the gene (i.e., huntingtin) that encodes for this protein (McColgan and Tabrizi, 2018; Roos, 2010). This gene is found on chromosome 4 in humans. HD has no known cure, despite the fact that its progression makes people totally reliant on others for everyday necessities, necessitating 24-h care (McColgan and Tabrizi, 2018; Roos, 2010). After being formerly believed to affect only motor neurons, it is now recognized as a multi-system disorder. Amyotrophic lateral sclerosis (ALS) mostly manifests as weakness, muscular atrophy, and ultimately paralysis (Rahimi et al., 2024; Masrori and Van Damme, 2020; Hou et al., 2019). This disease also becomes more severe than others because the affected individual does not survive after a few years (2–10). The most common cause of death is the failure of respiration due to severe muscle loss in the diaphragm. Due to this reason, the diagnosed patient is unable to breathe correctly. Also, while other diseases usually occur in the older generation, ALS could be diagnosed even in younger ages, such as in teenagers (Hardiman et al., 2017; Brown and Al-Chalabi, 2017). Other neurodegenerative diseases might exhibit unique pathologies but share commonalities in the progressive neuronal damage associated with aging.

### Aging and its role in neurodegeneration

A fundamental biological process, aging, raises the risk of illness and death by causing a deterioration in both physical and functional abilities. In terms of neurodegenerative illnesses, it is the most important risk factor (Azam et al., 2021). According to projections, the number of Americans over 65 will increase from 53 million in 2018 to 88 million by 2050, highlighting the growing prevalence of age-related illnesses. Neurodegenerative diseases stand out among these because of their significant effects on life expectancy and quality of life. Neurodegeneration is influenced by age in a number of ways. It is uncertain if aberrant protein aggregates like Aβ, hyperphosphorylated tau, and α-synuclein are directly linked to cognitive impairment, yet they are frequently found in older people’s brain tissue (Azam et al., 2021; Bourdenx et al., 2017). Neuroplasticity is also impacted by aging, increasing the brain’s susceptibility to genetic and environmental factors (Azam et al., 2021; Schaefers and Teuchert-Noodt, 2016).

The apolipoprotein E (APOE) ε4 allele, for instance, is a key gene that predisposes people to late-onset AD, and people with it exhibit structural brain changes well before cognitive symptoms appear (Azam et al., 2021; Erikson et al., 2016; Matteini et al., 2016).

### Bridging aging and neurodegenerative diseases

Neurodegeneration and aging constitute a continuum, with the onset and course of neurodegenerative illnesses being influenced by the characteristics of aging, such as loss of proteostasis (López-Otín et al., 2013), mitochondrial malfunction, and genomic instability (Azam et al., 2021). According to molecular research, aging of the brain may be an accelerated type of neurodegeneration, especially in extremely elderly people (Azam et al., 2021; Wyss-Coray, 2016). Additionally, developmental environmental exposures like trauma or poisons may have long-term consequences, making people more susceptible to neurodegenerative illnesses in later life (Nabi and Tabassum, 2022). The burden of neurodegenerative illnesses will rise sharply as the population ages, calling for immediate improvements in treatment approaches. To create interventions that can extend the lifespan of old individuals, it is essential to comprehend the biological processes of aging and how they interact with neurodegeneration. Addressing the underlying mechanisms of aging and neurodegeneration holds potential for more effective and long-lasting remedies, even though present treatments concentrate on managing symptoms.

### Current and emerging therapies for neurodegenerative diseases

Medication is frequently used to treat neurodegenerative diseases with the goal of reducing symptoms and delaying the course of the illness. To manage these issues, the following are some standard therapy options (Cascione et al., 2020; Lipton, 2004; Hallett and Standaert, 2004; Armstrong and Okun, 2020; Caraci et al., 2020; Song C. et al., 2022; Sudhakar and Richardson, 2019):

Inhibitors of acetylcholinesterase: For AD medications including galantamine, rivastigmine, and donepezil are commonly recommended to treat dementia-related symptoms. By blocking acetylcholinesterase, which facilitates better nerve-cell communication, these drugs may stabilize cognitive function and prevent future cognitive loss in AD patients.

Antagonists of NMDA receptors: Excessive NMDA receptor activation can lead to neurodegeneration in conditions like stroke, dementia, and neuropathic pain. By preventing glutamate, a neurotransmitter that can harm brain cells when it is hyperactive, memantine, a common NMDA receptor antagonist, helps control the medical indications of PD and AD (Lipton, 2004; Hallett and Standaert, 2004).

Agonists of dopamine: Dopamine agonists, which imitate the brain’s dopamine function, are crucial in the cure of PD because they help control symptoms such bradykinesia, rigidity, and tremors. Movement, memory, and other processes depend on dopamine, and agonists help make up for the loss of dopamine-producing neurons in Parkinson’s disease. Antipsychotic Drugs: These medications, which are frequently recommended for Parkinson’s and Alzheimer’s illnesses, can reduce neuropsychiatric symptoms like agitation, delusions, and hallucinations (Caraci et al., 2020). However, its use needs to be closely watched because of the possible adverse effects and elevated mortality risk.

Immunomodulatory substances: The goal of immunomodulatory therapies is to lessen inflammation in the brain, which is assumed to contribute to neurodegenerative illnesses (Song C. et al., 2022). Specific treatments aim to improve the brain’s ability to eliminate amyloid-β (Aβ), such as vaccinations and antibodies that target Aβ. In order to fight neurodegeneration, other researchers concentrate on the tau protein as a possible therapeutic target. Presently, immunotherapies are mainly in the developmental stage, although a few, such as lecanemab and Donanemab, have recently received approval for Alzheimer’s disease, but hold a promising future (Espay et al., 2024).

Gene therapy: By injecting new or altered genes into the brain, gene therapy is a cutting-edge method of treating neurodegenerative illnesses. Although for now gene therapies are considered an experimental approach but, in the future, this approach offers hope for long-term illness management and possible cures by correcting underlying genetic abnormalities, providing neuroprotection, and stimulating neurorestoration (Sudhakar and Richardson, 2019).

Pharmacological therapies for neurodegenerative diseases are challenging due to the limited efficacy of current drugs, progressive disease course and, more importantly, heterogeneity across motor, cognitive, and psychiatric domains. Despite ongoing research, there remains no definitive cure for these conditions, and the existing therapeutic options primarily focus on symptomatic relief. However, promising prospects for the future are emerging, fueled by advances in understanding the pathophysiological mechanisms underlying such medical conditions for the discovery of undiscovered pharmacological agents. Recent curative approaches have addressed the hallmark pathologies of neurological implications by concentrating on anti-amyloid and anti-tau therapies. Although these therapies have some potential, they cannot stop the development of neuronal degeneration or brain atrophy. Additionally, new pathways for intervention have been made possible by the development of advanced gene-modifying approaches like CRISPR/Cas9. Because they provide new opportunities for the selective genetic material modifications to rectify pathogenic processes at the molecular level, these cutting-edge techniques have shown promise in the treatment of a variety of aging disorders, containing AD, PD, and HD (Rahimi et al., 2024; Lamptey et al., 2022).

## Revolutionizing the treatment of aging-related neurodegenerative diseases: the promise of CRISPR-Cas gene editing

One of the most revolutionary and significant scientific breakthroughs, Clustered Regularly Interspaced Short Palindromic Repeats (CRISPR), was first discovered in the bacterial strain Escherichia coli in 1987 (Ishino et al., 1987). According to Jansen et al. (2002), CRISPR-associated (cas) genes were reported for the first time, while Makarova and Koonin (2006) later provided a comprehensive comparative genomic analysis and classification of these genes. Together, CRISPR and RNA-guided Cas proteins have been shown to function, and Barrangou’s team thoroughly verified the CRISPR-Cas system’s ability to protect prokaryotic cells against invasive phages in 2007 (Barrangou et al., 2007). Makarova et al. (2011) described that there are three main stages to the immunological response to CRISPR-Cas: adaptation, expression, and interference (Makarova et al., 2011). Multiple Cas-linked proteins make a group and form a complex that attaches itself to a particular DNA segment, frequently identified by a brief motif called PAM, and eliminates one portion of the nucleic acid, DNA known as the protospacer during the adaptation phase (Nuñez et al., 2014). The protospacer DNA is subsequently inserted as a space occupier into the CRISPR array by the adaptation complex, either by obtaining it from RNA through reverse transcription or by copying the repeat at the 5’ end (Swarts et al., 2012; Heler et al., 2015). The spacer section contains a specific sequence, and certain portions of the flanking repeats are present in each mature CRISPR RNA (crRNA), which is produced during the expression stage by transcription of the CRISPR array into pre-CRISPR RNA (pre-crRNA) (Brouns et al., 2008; Deltcheva et al., 2011). Based on the CRISPR-Cas variant, this processing is performed by non-Cas host RNases, a single multidomain Cas protein, or distinct components of a Cas complex. The Cas nuclease is guided to the protospacer or a comparable sequence in the genome of a virus or plasmid by the crRNA, which stays attached to the processing complex during the interference phase (Brouns et al., 2008; Jinek et al., 2012). The protospacer is then cleaved and rendered inactive. Based on the design principles of their effector modules, the CRISPR-Cas systems can be divided into two main classes along with six categories (I–VI) (Makarova et al., 2015). The basic difference between these systems is that the Class 1 (consisting of types I, III, and IV) uses multi-subunit effector complexes protein. On the other hand, the Class II system (consisting of types II, V, and VI) is comprised of large single effector proteins (Makarova et al., 2015; Charpentier and Doudna, 2013).

**FIGURE 1:**
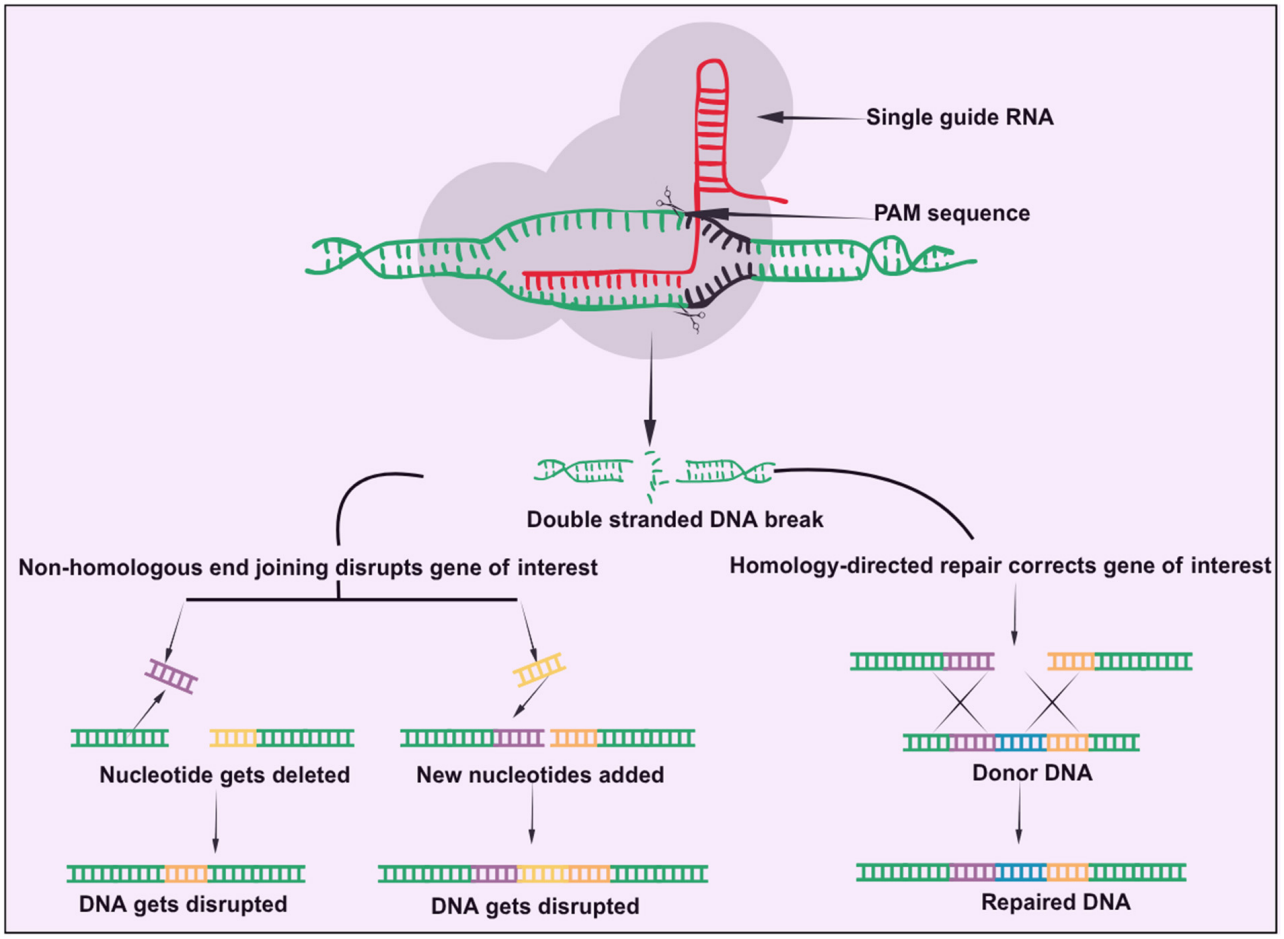
CRISPR-Cas9 mechanism of action. Bottom left-hand side of image shows non-homologous end joining while the bottom right hand side section shows the homology-directed repair.

Among the several CRISPR-Cas systems for genome editing, the Type II CRISPR-Cas9 system that is discovered from Streptococcus pyogenes (Spy-Cas9) is the most researched and extensively used (Jinek et al., 2012). For Spy-Cas9 to cleave DNA, two RNA molecules, i.e., a trans-activating crRNA (tracrRNA) and a crRNA, must be present, where these two RNAs merge to form a single guide RNA (sgRNA). In essence, the CRISPR-Cas9 framework comprises an RNA-directed Cas9 endonuclease coupled with a single guide RNA that precisely directs the Cas9 nuclease to designated loci within chromosomal DNA sequences. As a result, a double-strand break (DSB) is created, which can be restored by either the homology-directed repair (HDR) or the non-homologous end joining (NHEJ) pathway (Figure 1), which are both endogenous self-repair mechanisms contained within the organism (Cong et al., 2013; Mali et al., 2013; Chu et al., 2015).

A complicated structure with several domains, the Cas9 protein is divided into two separate sections referred to as the recognition (REC) lobe and the nuclease (NUC) lobe. A bridge helix (BH) with an elevated level of arginine residues connects these two lobes (Nishimasu et al., 2014). In order to allow the Cas9 protein to attach to the target DNA, the NUC lobe of Cas9 has a PAM-interacting domain in addition to two endonuclease domains, HNH and RuvC. Each of these two domains cuts a single strand of DNA, producing double-stranded DNA. Furthermore, SpCas9’s REC lobe is made up of many recognition domains (REC1-REC3) that let SpCas9 connect to DNA and RNA. Cas9 can function as a modifiable instrument to generate chromosomal double-strand cleavages *in vitro* and *in vivo* due to its strong and dependable activity (Jinek et al., 2014). As a result, CRISPR/Cas9 has evolved into a straightforward and adaptable RNA-guided genomic modification platform that can be implemented across diverse organisms and cellular populations, encompassing pigs, mice, rats, zebrafish, bacteria and human cells for the treatment of diseases (Hsu et al., 2014). Table 1 shows the comparison of different gene editing and gene regulation technologies, highlighting their respective advantages and limitations, but among these, the CRISPR/Cas9 has been widely adopted due to its ease in designing, cost-effectiveness and high efficacy. The foremost application of CRISPR/Cas9 in therapeutic applications occurred in 2016. Patients with advanced lung cancer were treated by a Chinese team using cells that had been altered using CRISPR/Cas9 (Cyranoski, 2016). Many clinical trials have been carried out in recent years, and a number of the results have been reported in published literature. These results include the application of CRISPR/Cas9-linked therapies for diseases like β-thalassemia, sickle cell disease (SCD), acquired immunodeficiency syndrome (AIDS), and various malignancies (Cyranoski, 2016; Frangoul et al., 2021; Xu et al., 2019).

**TABLE 1:** Comparative framework for genome editing approaches and regulation tools.

Gene editing method	Mechanism of action	Advantages (ADV)/limitations (LIM)
CRSIPR/Cas9	Produce double stand breaks by using cas9 guided gRNA at specific target site. In turn HDR and NHEJ repair mechanism gets activated and leads to knockout, correction or disruption of that target gene.	ADV: easy design, high efficacy, low cost. LIM: off targeting, less precise.
CRISPRi/CRISPRa/other dCas9-based activators and repressors etc.,	Alters target gene expression (down/up regulating) by using dead version of cas9 i.e., dCas9 fused with transcription activators/ repressors or epigenetic modifiers	ADV: reversible in nature, no permanent change in genome, safe, more specific. LIM: repeated dosing needed, incomplete knockdown (CRISPRi), limited by promoter context (CRISPRa).
Cas13 mediated RNA -editing/splice modulation/antisense oligonucleotides	RNA editing by RNA editing enzymes like Cas13, or by splicing to remove mutant exons	ADV: reversible in nature (antisense oligonucleotides), no permanent change in genome and directly modulate RNA splicing, stability and translation. LIM: only effect RNA, repeated dosing needed
Prime editing	It produces cuts only in one strand of DNA by the help of nCas9-RT fusion guided by the help of pegRNA at specific target site	ADV: highly precise, versatile, can install substitutions, insertions, deletions without DSBs. LIM: delivery challenges, larger and more complex machinery.
Base editing	This is for single base changes where cas9-deaminase complex is guided to the DNA site, Cas9 opens a small DNA bubble and deaminase acts on single base	ADV: highly precise for point mutations. LIM: limited to transition mutations only (not all possible edits), off targeting
Zinc finger nucleases (ZFN)	It uses Zinc finger proteins that recognize 3-bp DNA sequence. This method can be used to target the specific DNA sequence by kinking the fingers in the tandem. It uses Fokl endonucleases that dimerizes and creates DSBs	ADV: easy delivery due to compact and small size, proven in clinical trials, no guide RNAs needed. LIM: expensive, designing complexity and lower efficiency as compared to CRISPR
Transcription activator like effector nucleases (TALENs)	It uses TALE proteins that recognize single nucleotide. It uses Fokl endonucleases that dimerizes and creates DSBs	ADV: high efficacy and flexible target range LIM: lower efficiency as compared to CRISPR, delivery problem due to large size
RNA interference	It uses the siRNA/shRNA to degrade target mRNA in the cells.	ADV: easy design works in many organisms, reversible and transient in nature and effective in knocking down the gene expression. LIM: off targeting, efficiency varies across, incomplete knockdown

**FIGURE 2:**
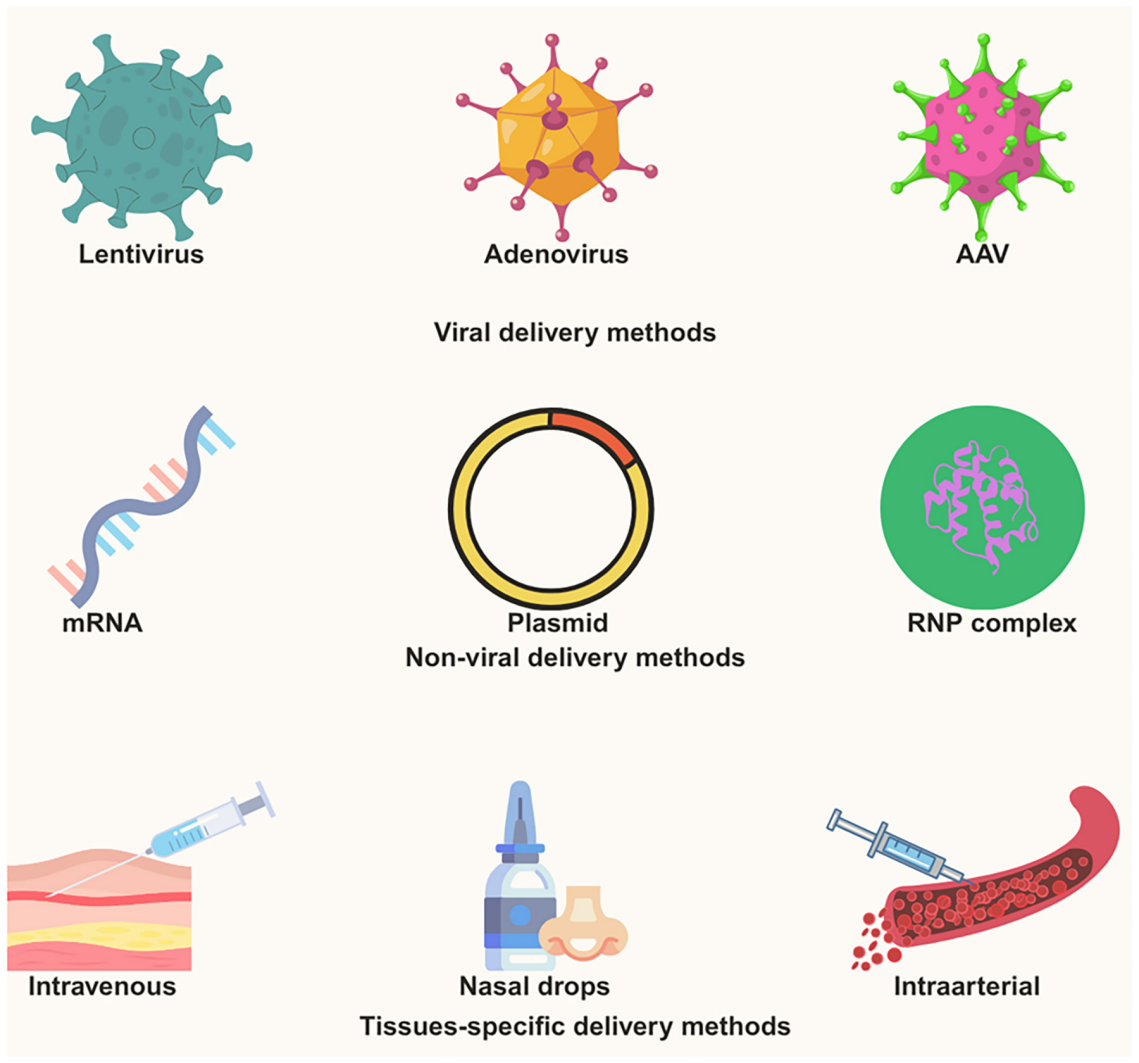
CRISPR-based delivery methods for neurodegenerative diseases.

The CRISPR components are usually injected into the cells to alter the genes in mammalian cells for therapeutic purposes. Mostly mRNA, viral, plasmid, as well as protein-based techniques are employed to transport the CRISPR/Cas9 elements within the cellular environments (Lino et al., 2018). Among these approaches, viral-mediated delivery represents the most employed strategy for CRISPR/Cas9 administration (Figure 2). To achieve this, a variety of virus delivery systems have been used that includes lentiviral vectors (LV), adenoviral vectors (AdV), and adeno-associated viruses (AAV) (Wang et al., 2017, 2019; Naldini, 2015; Daya and Berns, 2008). For the following reasons, they are frequently utilized in CRISPR genomic modification: The first benefit of using AAVs for illness treatment is their ability to penetrate the target cell and survive without the help of the host cell’s genome (Daya and Berns, 2008; Wang et al., 2019). Second, because of their varied capsids, AAVs can infect a range of tissues. The other advantages of using AAVs also include tissue tropism, episomal persistence in non-dividing cells, and low genomic integration rates (Bulcha et al., 2021).

Introducing Cas9-encoded mRNA into cells is another popular technique for introducing CRISPR technology. mRNA-based methods minimize the possible hazards associated with becoming an integral part of the host genome and serve a transient purpose. Furthermore, there is a rapid translation of Cas9 mRNA into protein; these tactics offer the benefit of quicker outcomes.

Another appealing technique for introducing CRISPR components into cells is plasmid-based methods. This strategy has several advantages. First, the method of gene synthesis is simple. Second, continuous expression is possible since the generated gene can be delivered to the target cell via a plasmid without needing to integrate into the host genome (Lino et al., 2018).

**FIGURE 3:**
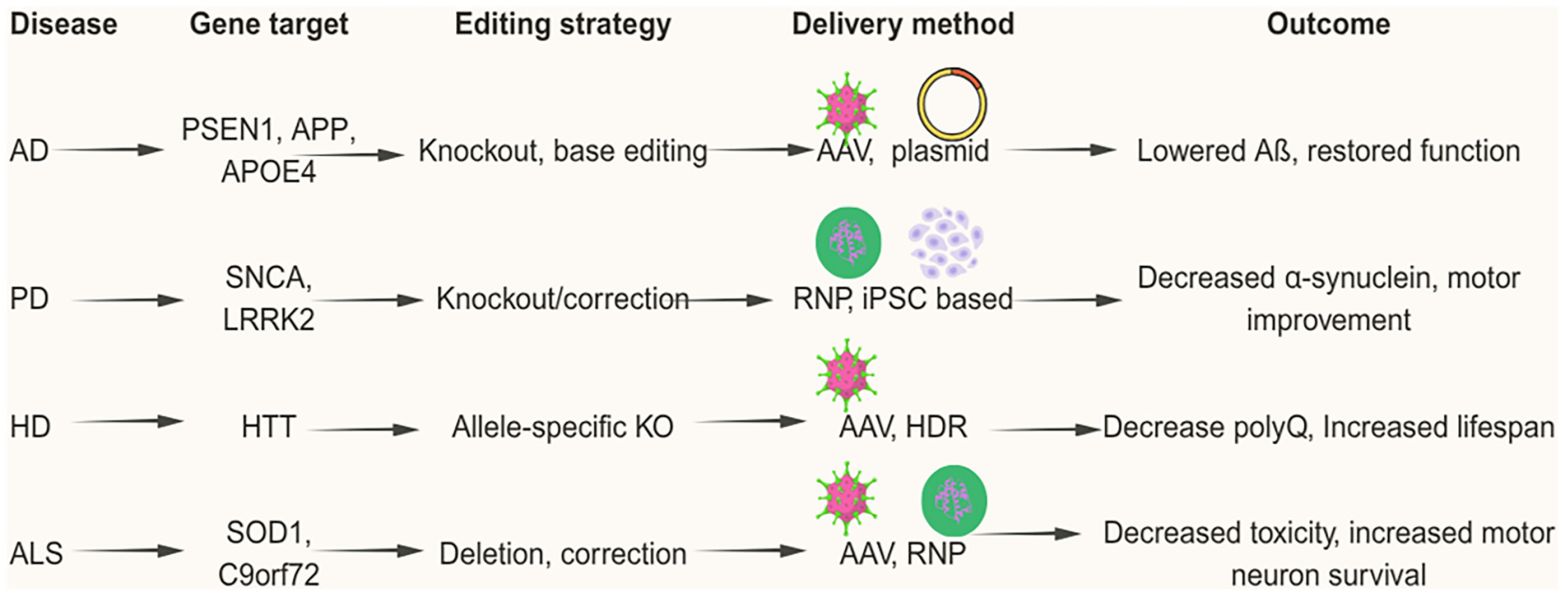
CRISPR/Cas9 applications across major neurodegenerative disorders.

Genetic engineering has been revolutionized by protein-based CRISPR/Cas9 techniques, which are another efficient way to deliver CRISPR components. Ribonucleoprotein (RNP) is a crucial part of the CRISPR/Cas9 mechanism in this system. The RNP complex, which is made up of the Cas9 protein and an sgRNA, is how the Cas9 protein is transported to the target cells in the CRISPR-Cas9 RNP delivery system (Kim et al., 2014). Enhanced selectivity, diminished unintended consequences, and enhanced performance are just a few of the benefits that make the RNP complex an appealing delivery technique. Immune cells, primary cells, and stem cells are among the various model animals and cell types that the Cas9 ribonucleoprotein (RNP) method can be used to with ease (Kim et al., 2014; Zuris et al., 2015; Yin et al., 2016). In the therapeutic intervention for neurodegenerative illnesses, the CRISPR-Cas9 system has shown encouraging outcomes. The CRISPR-Cas9 applications that have been used to treat various illnesses will be discussed in the sections that follow, and a comparative mechanism is shown in Figure 3.

### Alzheimer’s disease (AD)

AD one of the leading causes of progressive neurological illness that is currently a leading cause of death and disability, behind cancer and heart disease. Memory impairments and cognitive decline are common features of the disease, which eventually affect language, behavior, movement, reasoning, judgment, and memory before resulting in dementia and mortality (Querfurth and LaFerla, 2010). The cleavage of amyloid precursor protein (APP) generates Aβ peptides, which accumulate to form amyloid plaques–abnormal deposits implicated in the pathogenesis of AD (Querfurth and LaFerla, 2010; Selkoe and Hardy, 2016). Neurofibrillary tangles (NFTs) are another constituent of these plaques, which, together with Aβ are responsible for the AD symptoms. These are the two neuropathological features that most commonly define AD. The classical hallmark of AD is thought to be the link between these two pathologies. According to researchers, the conventional Aβ hypothesis offered a fundamental basis for the creation of possible AD modulating treatments that target and stop the production of Aβ and encourage the removal of harmful proteins from the brain, including Aβ (Du et al., 2018). There have been several unsuccessful trials to create disease-modulating therapies utilizing *in vivo* disease models of the illness, nevertheless. As a result, the CRISPR/Cas9 technique has become more well-known in the field of AD in recent years because of its low consumption and brief trial period. For activities including creating AD models, detecting harmful genes through screening, and implementing targeted therapy, it is currently widely used.

A tiny proportion of AD cases are familial, also referred to as familial AD or FAD, even though most cases are sporadic (Bekris et al., 2010). These instances arise due to pathogenic variants inherited in an autosomal dominant manner affecting one of the following loci: the gene encoding amyloidogenic precursor protein (APP), presenilin-1 (PSEN1), or presenilin-2 (PSEN2). The main cause of FAD is PSEN1 mutations, which usually cause symptoms to appear earlier than those caused by mutations in the other two genes (Bekris et al., 2010). The majority of PSEN1 mutations result in an elevated production of the more aggregation-prone Aβ42 compared to Aβ40. The development of Aβ plaques in the brain, a defining feature of Alzheimer’s disease, is known to be facilitated by this aberrant Aβ42 synthesis (Bateman et al., 2012; Sherrington et al., 1995). Recent research suggests that autosomal dominant mutations may be successfully corrected using the CRISPR/Cas9 technology. This nucleic acid alteration tool’s potential for genetic modification is further supported by reports that it has effectively corrected similar kinds of mutations. Later research has shown that employing the CRISPR-Cas9 genome editing technique modifies cell models derived from PSEN2 N141I variant-carrying patient fibroblasts can effectively reverse related electrical activity disruptions and reestablish a physiological Aβ42 to Aβ40 ratio (Sherrington et al., 1995; Haass and Selkoe, 2007; De Strooper, 2007). These findings were further supported by other research investigations that used iPSCs obtained from patients and CRISPR/Cas9 to fix the genetic alterations in the PSEN gene in FAD. Furthermore, another study showed that the background of endogenous γ-secretase was abolished when PSEN1 genes were knocked out in N2a cells using the Type II CRISPR/Cas9 method. Additionally, they found that the synthesis of Aβ42 and Aβ40 was reduced when the PSEN1 mutation-derived recombinant protein was introduced (Pimenova and Goate, 2020).

When Swedish APP (APPswe) mutations were eliminated using Type II CRISPR/Cas9 technology, the expression of Aβ protein decreased, according to a distinct study conducted on patient-derived fibroblasts (György et al., 2018). This mutation, often referred to be Swedish KM670/671NL APP, raises the amounts of Aβ protein by increasing enzymatic cleavage through β-secretase. Furthermore, Guyon led a team of researchers that introduced a novel mutation in 2021 by modifying the APP gene using this technique (Guyon et al., 2021). In both mammalian cell lines (SH-SY5Y cells and HEK293T cells) containing the APP gene, having the amine group removed from the cytosine one and the cytosine two R groups. They changed the alanine codon to threonine (Guyon et al., 2021). According to their reported findings, they succeeded in the insertion of the A673T mutation in every 53 out of 100 HEK293T cells, along with a unique mutation in the amino acid residue number 674, which was replaced from E to K. This alteration further reduced the accumulation of Aβ peptide (Guyon et al., 2021).

It is now known that the APOE (apolipoprotein E) gene increases a person’s risk of developing sporadic AD (SAD). In AD patients, the APOE allele primarily originates from an individual’s central nervous system, containing an astrocyte-rich area. There are several variations of the APOE gene, such as E2, E3, and E4. Among these variations, the strongest genetic risk factor for SAD is the APOE4 type (Lozupone et al., 2023). To ascertain the function of APOE4, Lin and associates used hiPSC and the Type II CRISPR system in 2018. The research-driven outcomes of their study provided the conclusion that, depending on the kind of cell, APOE4 had different effects on Aβ metabolism (Lin et al., 2018). Additionally, Wadhwani et al. (2019) investigated possible APOE4 treatment targets in 2019. They corrected the E4 allele to the E3/E3 genotype in iPSCs from two AD patients using the Type II CRISPR system. The findings demonstrated that E3 neurons exhibited lowered numbers of phosphorylated tau protein (Wadhwani et al., 2019). Their study thus also suggests that E3 neurons may be more resistant to ionomycin-driven cellular toxicity in comparison to the E4 neurons.

### Parkinson’s disease (PD)

Without any conflict, PD has now taken the place of the second most common neurodegenerative disorder (Jankovic, 2008; Kalia and Lang, 2015). Like AD, this disease also primarily occurs due to aging and affected individuals are of 55-plus aged. Impaired body movements are a defining feature of these diverse neurodegenerative disorders with medical implications (Jankovic, 2008; Kalia and Lang, 2015). As we all know, dopamine is one of the most important neurotransmitters found in humans, playing a crucial role in controlling normal muscular movements. These signal transmitter molecules are produced in the section of the brain known as the substantia nigra pars compacta (and abbreviated as SNPC) (Kalia and Lang, 2015). In PD, the SNPC region fails to produce adequate levels of dopamine, which, over time, as the individual ages, continues to decrease. Now that the dopamine levels are continuously declining in the patient, normal muscular movements turn out to be impaired, resulting in visible abnormal movements (Kalia and Lang, 2015; Surmeier et al., 2017; Dorsey et al., 2018). According to Blesa and Przedborski (2014), this sudden decrease in dopamine count in the striatum is significantly reduced. This reduction impairs the motor circuit’s ability to function, which ultimately causes PD symptoms. From reported symptoms and available literature, we know that such symptoms may include resting tremors (unwilling shaking gestures without any apparent will), bradykinesia (slowness in locomotory speed and actions), and rigidity (increased resistance to arm and leg movement) (Blesa and Przedborski, 2014; Hacker et al., 2012).

Another distinguishing feature is the existence of intra-cytoplasmic Lewy bodies (LB), which are mainly made up of ubiquitin and α-synuclein (Blesa et al., 2012). Although mutations in the α-synuclein gene have only been associated with rare familial cases of Parkinson’s disease, it is important to remember that all Lewy bodies include α-synuclein. The remaining 10% of PD patients have familial PD brought on by mutations in particular genes such as PRKN/PARK2, SNCA, PINK1, UCH-L1, LRRK2, PARK7, MAPT/STH, and DJ-1, GBA. Around 90% of patients having PD have no known etiology (idiopathic). These mutations might possibly be linked to sporadic Parkinson’s disease (PD) (Cota-Coronado et al., 2020; Nalls et al., 2019). The SNCA gene is intimately associated with α-synuclein expression (Ferreira and Massano, 2017). SNCA gene mutation, particularly at amino acid residue number 53, is one of the most significant risk factors of PD. At this position, originally lies Alanine residue, which gets replaced by Threonine due to the occurrence of a missense mutation. Although SNCA contains several mutations, A53T stands out due to its correlation with Parkinson’s disease (Spira et al., 2001). According to a study done in the year 2022 by Yoon et al. (2022), the increase in α-synuclein protein and reactive microgliosis caused by it or PD-linked motor neuron indications may be considerably improved by employing the newly developed CRISPR-Cas9 technique by targeting this mutation within the SNCA gene.

Several attempts have been made to test whether this technique is effective against PD. An example of such an effort is Kantor et al. (2018), for utilized CRISPR-Cas9 technology; their team chose a lentiviral vector. They succeeded and reported that using the CRISPR-Cas9 technique, mRNA linked to the SNCA gene was precisely downregulated, limiting its protein expression (Kantor et al., 2018). Further attempts were made by Chen et al. (2021), who sought to identify the mechanism that works behind the functioning of the SNCA gene within the nucleus of a cell. For this purpose, their group obtained human-induced pluripotent stem cells from patients diagnosed with PD having the A53T and SNCA-triplication dominant mutations, along with their version of the same gene, which was obtained upon modification using the CRISPR-Cas9 technique. In their findings based on preclinical models, it was reported that the absence of this gene leads to the development of resistance against Lewy body pathology (Chen V. et al., 2020; Chen X. et al., 2020). Then Zhou et al. (2015) tried to test the same using porcine models obtained from a domestically grown environment. Then, a combinatory technique encompassing CRISPR-Cas9 and somatic cell nuclear transfer (abbreviated as SCNT) was used to determine the effects of PARK2 and PINK1 genes (Zhou et al., 2015). Both of these genes were then knocked out with a success rate of approximately 38%. In a separate notable study focusing on nigral dopaminergic neurons (DN), CRISPR/Cas-mediated ablation targeted the ATP13A2 (PARK9), DJ-1 (PARK7), and PRKN (PARKIN) genes. Integrated transcriptomic and proteomic analyses across these isogenic cell models consistently identified oxidative stress as a common dysregulated pathway (Ahfeldt et al., 2020). In another more recent study, it has been reported that loss-of-function mutations in DNAJC6, which encodes the HSP40 co-chaperone auxilin, are associated with early-onset PD pathogenesis. To elucidate the functional consequences of such genetic alterations, CRISPR-Cas9-mediated genome editing was applied to human embryonic stem cells (hESCs). Transcript profiling and experimental validation suggested that disruptions in DNAJC6 dependent endocytic processes impair WNT-LMX1A signaling during the development of dopaminergic neurons found within the midbrain (mDA). Such impairments cause the reduced LMX1A expression throughout the process of neurogenesis, which may consequently lead to the generation of developmentally compromised mDA neurons. mDA neurons are known to exhibit increased pathogenic vulnerabilities (Wulansari et al., 2021; Nouri Nojadeh et al., 2023).

### Huntington’s disease (HD)

An autosomal dominant pattern of inheritance leads to a single genetic mutation that causes Huntington’s disease (HD), a progressive neurological illness. According to Bates et al. (2015), it is the most common hereditary neurodegenerative illness, and it is caused by a pathogenic mutation [CAG trinucleotide repeat expansion, encoding glutamine (Q)] (Paulsen, 2011). As a result, the huntingtin protein’s N-terminal domain develops an extended polyglutamine strand (Kim et al., 2001; Orr and Zoghbi, 2007). A wide range of molecular and cellular processes in the brain are disrupted by this mutant protein, which leads to clinical symptoms like a continuous decrease in decision-making capabilities, accompanied by multiple disorders such as chorea, dystonia, non-coordination, and psychiatric disorders (Waldvogel et al., 2015; Byun et al., 2022). HD is a suitable candidate for gene therapy since it is caused by a single genetic alteration, accompanied by the deposition of faulty protein aggregates. Nowadays, with advancements in the CRISPR/Cas9 technique, it is also pronounced as one gene one therapy and has the potential to inhibit the expression of the faulty HTT gene (Shin et al., 2016).

Shin and associates carried out a study to increase allele specificity. They employed a customized allele-selective CRISPR/Cas9 approach based on SNPs that modify the Pro-tospacer Adjacent Motif (PAM) (Shin et al., 2016). This approach combined a thorough understanding of the huntingtin (HTT) gene haplotype structure with a focus on patient-specific CRISPR/Cas9 locations. According to them, the objective is to specifically deactivate the mutated HTT allele and limit its conversion into a specific diplopic gene (Shin et al., 2016). Furthermore, Suzuki and colleagues devised a method known as homology-independent targeted integration (HITI), which employs CRISPR to effectively eliminate nucleic acids in a growing population of cells observed through *in vitro/vivo* methods (Suzuki et al., 2016). By enhancing visual function, HITI was demonstrated to be effective in a rat retinitis pigmentosa model, a disorder that results in retinal degeneration (Suzuki et al., 2016). Yang et al. (2017) showed in another study that polyglutamine expansion-driven toxic environment within neuronal cells within the mature brain may be successfully and permanently eliminated by employing CRISPR. They came to such conclusions upon performing their studies on the HD140Q-KI mouse model, where HTT was eliminated. CRISPR/Cas9 in HD140Q-KI mice to deplete HTT in a non-allele-specific way (Yang et al., 2017). According to them, the experimental group showed a significant improvement in motor dysfunction and a notable decrease in reactive astrocytes (Yang et al., 2017). Additionally, a study by Ekman et al. (2019) showed that neurotoxic inclusion development was reduced by two times when CRISPR-Cas9 was used for targeting the altered HTT gene in an R6/2 mouse model strain. The mouse model used by them carried exon 1, which is found on the HTT gene, having 115–150 CAG repetitions. In the same mice, this also resulted in a longer lifespan and improvement in some of the motor neuro defects (Nouri Nojadeh et al., 2023; Xie et al., 2019). Such outcomes show the potential of CRISPR-Cas9 as a tool for HD, reaffirming its applicability to address other similar neurodegenerative diseases.

### Amyotrophic lateral sclerosis (ALS)

Also, named as Lou Gehrig’s disease (given upon a famous athlete), it affects the human motor system and progresses quickly. According to van den Bos et al. (2019), this disorder is brought on by the central nervous system’s motor neuron. Dysfunction in motor neurons is the most observed pathological hallmark affecting the whole-body movement and is the most frequently observed type of motor neuron disease (MND). As the disease progresses, patients witness multiple malformations encompassing lack of strength, severe muscle loss (atrophy), partially or complete lack of movement (or paralysis), and finally failure in respiration due to weakened diaphragm muscles, becoming the most common cause of death because of this dysfunction (Oskarsson et al., 2018). There are two forms of ALS: the first one that runs in families, familial ALS (fALS), which makes up only a small portion of the observed cases (approximately 10%), while the other one, which is developed during an individual’s lifetime due to unclear reasons (Boylan, 2015). C9orf72, SOD1, TARDBP, and FUS are the most common pathogenic genes linked to ALS (Bursch et al., 2019). Furthermore, some of the most frequently observed reasons for ALS patients, both inherited (40%) and sporadic (5-6%), are the recurrence in noncoding regions of the hexanucleotide sequence of the gene C9ORF72 (DeJesus-Hernandez et al., 2011; Majounie et al., 2012). To eliminate HRE from the C9ORF72 chromosomal locus, Meijboom’s team used an adeno-linked viral vector method for the transfer of the CRISPR-Cas9 system. Various models were used to successfully demonstrate this purpose, including murine models, patient-derived iPSC motor neurons, organoids, and primary cortical neurons. All-important markers C9-ALS/FTD (such as RNA foci, poly-dipeptides, and haploinsufficiency) used in ALS detection and treatments were observed to lower signifying positive results. Treating these illnesses with this therapy approach is hopeful (Meijboom et al., 2022).

In a work by Deng et al. (2021), they edited transgenes (hSOD1-G93A) linked to Lou Gehrig’s disease using transgenic mice with CRISPR/Cas9. In two other mouse model studies with hSOD1-G93A (G1H and G1L) mutations, it was shown that the gene-editing approach is beneficial in targeting and correcting the mutation. Another work addressed the SOD1 E100G mutation by using the CRISPR/Cas9 technique. iPSCs from an ALS patient having these mutations underwent targeted gene repair (Yun and Ha, 2020). Later, the iPSCs developed into motor neurons. In addition, Chen et al. (2021) described a quick, easy, and effective method for CRISPR-Cas9-induced point mutations associated with ALS in human iPSCs without the need for antibiotic selection.

## Spinocerebellar ataxia

In 2018, Soong and Morrison (2018) state that spinocerebellar ataxias (SCAs) constitute a category of progressive neurodegenerative conditions that mostly affect the cerebellum and are transmitted in an autosomal dominant fashion. The main clinical characteristic of SCAs is a progressive decline in stability and agility, which is frequently concomitant with communication difficulties. Observable features typically manifest in adulthood. To date, over 40 genetically different subtypes of SCAs have been found, making them a diverse collection of illnesses. Every subcategory is identified by the abbreviation SCA, which is subsequently succeeded by a numerical sequence that reflects the episode in which the causal gene or disorder locus was identified (Maas et al., 2015). More than half of cases are of the standard and well-defined subtypes SCA1, SCA2, SCA3, and SCA6, while the remainder instances are of uncommon variations (Jacobi et al., 2018). The SCAs can be divided into two primary groups in terms of genetics: those resulting from non-repeat mutations and those resulting from dynamic repeat expansion mutations. The prevalence of neurological disorders that are not classified as SCAs is also significantly influenced by dynamic repeat expansions (Maas et al., 2015). Repeat expansion mutations are the cause of at least 12 SCAs. Six of these mutations [specifically SCA1, SCA2, SCA3/Machado-Joseph disease (SCA3/MJD), SCA6, SCA7, and SCA17], result from pathogenic CAG trinucleotide repeat amplifications within coding sequences that produce extended polyglutamine tracts in the respective disease-associated proteins. These illnesses are therefore known as polyglutamine SCAs (Durr, 2010; Paulson et al., 2017).

The main goal of reducing mATXN3 in research that used the CRISPR technique for SCA3 was successfully achieved by removing the mutant trinucleotide CAG expansions within the ATXN3 locus (Ouyang et al., 2018). This was accomplished through targeted disruption of the pathogenic CAG trinucleotide repeats located within the tenth exon’s region or at the beginning of 11 exon (Ouyang et al., 2018). The gene was primarily repaired by the NHEJ process, which ultimately produced a shortened ATXN3 incorporating a premature termination signal at the initiation of the eleventh exon sequence. He et al. (2021) also showed that the 74-repeat CAG trinucleotide extension present in exonic segment 10 of ATXN3 in SCA3-iPSCs can be successfully repaired using dual sgRNA/Cas9n nickase approach combined with homologous recombination methodology in an alternative investigation utilizing CRISPR/Cas9 genomic modification technology for accurate genetic correction through HR-mediated repair and complementary single guide RNAs. This results in a targeted and effective attenuation of aberrant ATXN3 protein expression. Furthermore, Song G. et al. (2022) developed efficient methods for one-step genetic repair in SCA3-iP-SCs of the SCA3 patients by utilizing homologous recombination in conjunction with a CRISPR/Cas9 technology. By creating SCA3 illness models in particular neurons that were differentiated based on the cerebellar area and disease-specific features, they further advanced their research. A recent study successfully developed and validated a CRISPR/Cas9 treatment strategy for fibroblasts derived from SCA1 patients (Pappadà et al., 2022). The method effectively reduced the synthesis of both healthy and mutant ATXN1 protein by utilizing G3 and G8 guide RNA/Cas9 ribonucleoprotein assemblies. This research demonstrates the encouraging results in preclinical models of some polyQ-related disorders utilizing the CRISPR genomic modification technique; however, the therapeutic applicability may vary across its subtypes due to genetic content (Pappadà et al., 2022).

## Future directions, limitations and conclusion

Even though research into aging and neurodegenerative diseases has come a long way (Shamsi et al., 2024, 2025; Shahwan et al., 2024; Hassan et al., 2025; Alrouji et al., 2025), we still don’t have treatments that can truly prevent or reverse these conditions. One of the biggest challenges is that we lack effective disease models that can accurately mimic what happens in the human brain during neurodegeneration. In this review, we’ve highlighted several key features of aging, like DNA damage, cellular senescence, and mitochondrial dysfunction, that are closely tied to these diseases. Going forward, it will be crucial to build better models that reflect how these aging-related changes contribute to conditions like AD and PD and other neurodegenerative diseases.

One of the most promising developments in recent times is the rise of CRISPR/Cas9 gene editing (Cong et al., 2013; Hsu et al., 2014; Nouri Nojadeh et al., 2023; Talat et al., 2025). This technology gives scientists the ability to precisely edit genes linked to neurodegenerative diseases (Table 2) and opens the possibility for targeted clinical therapies. So far, early studies in animals have shown promising results, but before we can bring these advances into human medicine, there are still significant hurdles to overcome. Two examples of such ongoing CRISPR-mediated clinical trials in humans include EDIT-101 (Pierce et al., 2024) (Leber Congenital Amaurosis, LCA) and NTLA-2001 (Gillmore et al., 2021) (Transthyretin (ATTR) Amyloidosis).

**TABLE 2:** Preclinical Clustered Regularly Interspaced Short Palindromic Repeats (CRISPR) interventions in neurodegeneration.

Disease	Editing method/tool	Delivery system used	Efficacy/behavioral changes/key outcomes
Alzheimer’s disease	CRISPR/spCas9	Amphiphilic RNP complexes	45% indels, 34% reduction Bace1 mRNA, Positive improvements (Park et al., 2019)
Alzheimer’s disease	CRISPR RNA-guided Adenine Base editors/NG-ABE8e	AAV9	Rescued cognitive decline; improvements in tau pathology and behavior (Gee et al., 2024)
Alzheimer’s disease	CRISPR-Cas9 via sgRNA	AAV9-Cas9-SW1	Decrease in Aβ pathology, reduced microgliosis, neurite dystrophy, cognitive improvements (Duan et al., 2021)
Alzheimer’s disease	CRISPR/saCas9	AAV	Editing ameliorates neuropathologic, electrophysiologic, and behavioral deficits in an AD knocking mouse model (Aulston et al., 2024)
Parkinson’s disease	dCas9-DNMT3A (CRISPRi/epigenetic silencing)	Engineered exosomes with targeting peptide (RVG), delivered across BBB via focused ultrasound; sgRNA + dCas9-DNMT3A complex delivered via the exosome.	Motor deficits rescued, balanced, reduced α-synuclein expression, rescued apoptosis, slowed disease progression (Kong et al., 2024)
Parkinson’s disease	Cas9 (SaCas9-KKH) + sgRNA targeting the mutant SNCA allele (A53T); direct deletion/suppression of mutant SNCA	AAV-DJ (AAV serotype DJ)	Motor deficits rescued, Reduced α-synuclein accumulation, reduced neuroinflammation (microgliosis), protected dopaminergic neurons (Yoon et al., 2022)
Parkinson’s disease	dCas9-activator system (CRISPRa)	Lentiviral constructs: N-dCas9 (SpCas92–573) fused to DnaE-N-Intein and C-dCas9-(SpCas9574–1368) fused to DnaE-C	Striatal astrocytes were converted into GABAergic neurons which integrate into striatal circuits and partial rescue of voluntary motor behavior deficits (Giehrl-Schwab et al., 2022)
Huntington’s disease	CRISPR/spCas9	AAV split system	HTT protein reduction (10%–80%), motor deficits rescued (Yang et al., 2017)
Huntington’s disease	CRISPR-Cas9 via sgRNA	AAV	∼50% decrease in neuronal inclusions; improvement in motor symptoms; increased life span (∼15%) relative to control (Ekman et al., 2019)
Huntington’s disease	CRISPR-Cas9/lentiCRISPRv2	AAV8	Identified genes (DNA repair modifiers) that influence the rate of somatic CAG expansion (Mouro Pinto et al., 2025)
Huntington’s disease	CRISPRi (dCas9-sgRNA)/LentiCRISPR v2 plasmid	Transduction	Delayed motor deterioration; improved locomotor activity; behavioral outcomes improved in treated mice vs controls (Seo et al., 2023)
Amyotrophic lateral sclerosis	CRISPR/SaCas9	AAV9	65% reduction in SOD1 protein, Motor deficits, muscular strength and survival rescued (Gaj et al., 2017)
LCA10	CRISPR/SaCas9	AAV5	27.9 % and 21.4% indels (Maeder et al., 2019)
Fragile X syndrome	CRISPR/spCas9	CRISPR-Gold RNP complexes	14.6% indels, ∼50% reduction in mGluR5 protein and mRNA levels, rescued repetitive jumping and excessive digging (Lee et al., 2018)
Retinitis pigmentosa	HDR/SpCas9 + sgRNA-MS2 + RecA-MS2	Plasmid electroporation	2% gene correction, Partial rescued (Cai et al., 2019)
MECP2 duplication syndrome	CRISPR/SpCas9	AAV split system	Reduction on MECP2 protein by around 50% and improvements in social recognition (Yu et al., 2020)
Hearing loss (DFNA36)	CRISPR/SpCas9	Cationic lipid mediated RNP complexes	1.8% indels, stability in auditory responses (Lee et al., 2018)

Gene therapy, while full of promise, also comes with its own set of challenges. CRISPR based gene editing technologies have opened new vistas in the treatment of neurodegenerative diseases (Table 2), but they have confronted several critical challenges that include the foremost concerns of off target effects that result in unintended editing in the genome resulting in deleterious mutations (Lopes and Prasad, 2024; Guo et al., 2023) or the delivery problem due to its large size that makes inefficient delivery across the blood brain barrier (Zou et al., 2022). Although recent advancements in assessment of CRISPR-induced off-target editing (*in silico* methods and experimental methods) and approaches to reduce off-target genome editing (Improvement of nucleases, sgRNAs, DSB-independent editing, Anti-CRISPR proteins and delivery methods for spatiotemporal control of editing) have been made, yet off-target activity remains a significant safety consideration in the clinical setting (Guo et al., 2023; Lopes and Prasad, 2024). Additionally, the difference between temporary or transient (CRISPRi and CRISPRa, Prime editing, Base editing, etc.) and permanent CRISPR-derived gene editing should be considered (Pacesa et al., 2024). The transient approaches are usually reversible, but they are safer, cost-effective, fast and have less off-targeting effects as compared to permanent and traditional CRISPR-mediated DNA editing (Pacesa et al., 2024). The other main limitation is that when CRISPR/Cas9 induces DNA damage in p53-proficient cells, it leads to its activation in turn as the safety response that eventually leads to cell cycle arrest and diverts the cells toward cell apoptosis, hence the CRISPR-induced gene editing efficacy is compromised (Haapaniemi et al., 2018). Another special aspect to consider is the cell type specificity; for example, unintended gene editing in the CNS (central nervous system) could lead to unpredictable and lethal outcomes, hence the current research is focusing on exploring more specific delivery systems and CRISPR effectors with selective tropism to enhance precision. The other things that can’t be ignored while designing the effective CRISPR system against diseases includes the check on preexisting immune response (humoral and cell mediated adaptive) against Cas9, as it has been discovered that many people already have antibodies and T cells against the cas9 (spcas9 and sacas9) (Shen et al., 2022; Charlesworth et al., 2019) and also CRISPR therapy has been reported to cause several immunogenic reactions that is considered its major setback in the field of gene therapy (Hakim et al., 2021; Ewaisha and Anderson, 2023).

Moreover, specifically considering CRISPR to treat neurodegenerative diseases, the fundamental limitations like the sporadic nature of neurodegenerative diseases (Zhang et al., 2020; Bertram and Tanzi, 2012; Nouri Nojadeh et al., 2023). Also come across as CRISPR is only applicable to target rare familial forms of neurodegenerative diseases with known monogenic causes, which account for a small subset of patient cases (Nouri Nojadeh et al., 2023). Apart from this, there are several ethical and regulatory hurdles that must be considered, including patient consent, long-term safety monitoring and germline editing.

**FIGURE 4:**
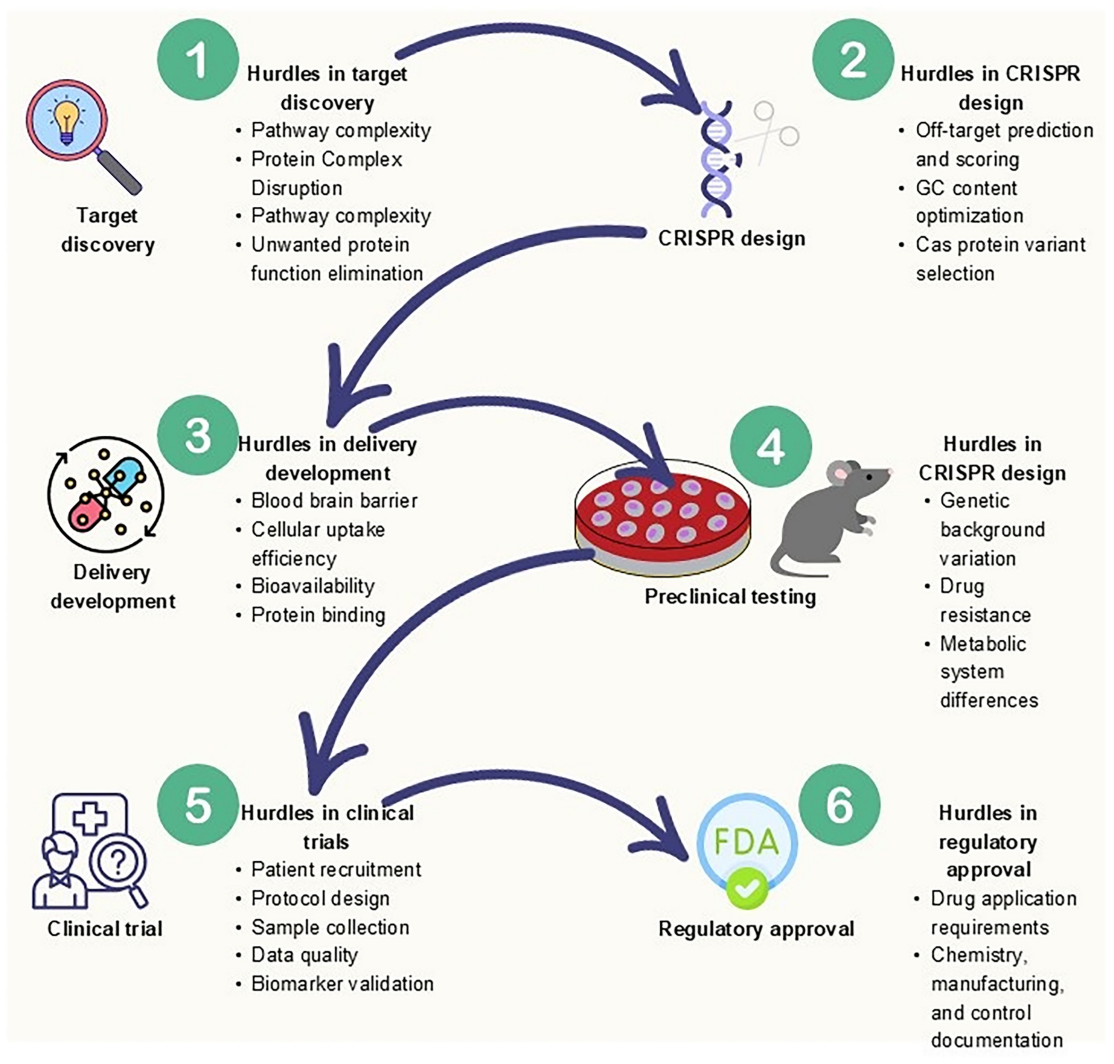
Future roadmap: from CRISPR edits to clinical therapy.

Looking ahead, the path to effective treatments for neurodegenerative diseases will depend on how well we can bring together what we know about aging, genetics, and advanced technologies (Figure 4).

Some key steps include:

- Creating better models that capture both aging and disease characteristics.
- Carefully selecting gene targets that work in harmony with other biological systems.
- Improving how we deliver gene editing tools, while minimizing side effects.
- Exploring combined approaches that bring gene therapy together with drugs or other treatments.

While the road ahead is still long, the progress being made gives us hope. With continued research and collaboration, we’re getting closer to developing therapies that can make a real difference for millions of people living with these devastating diseases.
