# Correction: CRISPR-Cas9: bridging the gap between aging mechanisms and therapeutic advances in neurodegenerative disorders

**DOI:** 10.3389/fncel.2025.1739705

**Published:** 2025-11-24

**Authors:** Anas Shamsi, Mohammed Alrouji, Othman AlOmeir, Syed Tasqeruddin, Khuzin Dinislam, Azna Zuberi

**Affiliations:** 1 Centre of Medical and Bio-allied Health Sciences Research, Ajman University, Ajman, United Arab Emirates; 2 Department of Medical Laboratories, College of Applied Medical Sciences, Shaqra University, Shaqra, Saudi Arabia; 3 Department of Clinical Pharmacy, College of Pharmacy, Shaqra University, Shaqra, Saudi Arabia; 4 Department of Pharmaceutical Chemistry, College of Pharmacy, King Khalid University, Abha, Saudi Arabia; 5 Department of General Chemistry, Bashkir State Medical University, Ufa, Russia; 6 Department of Obstetrics and Gynecology, Northwestern University, Chicago, IL, United States

**Keywords:** CRISPR-Cas9, gene editing, neurodegenerative diseases, Alzheimer's disease, Parkinson's disease, Huntington's disease, amyotrophic lateral sclerosis (ALS), aging

In the published article, in the author list, the fifth author's name was erroneously spelled as “Khzuin Dinislam” instead of the correctly spelt version “Khuzin Dinislam”.

The original version of this article has been updated.

